# Styloid process-related internal carotid artery dissection: extensive literature review of diagnosis, treatment and outcomes (exemplified with a single-center case series)

**DOI:** 10.1007/s00234-025-03616-y

**Published:** 2025-04-26

**Authors:** Tomas Klail, Ludwig Sachs, Leonidas D. Panos, Oliver Y. Urban, Teresa Siller, Sara Pilgram-Pastor, Roland Giger, Martin Müller, Franca Wagner

**Affiliations:** 1 University Institute of Diagnostic and Interventional Neuroradiology, Inselspital, Bern University Hospital and University of Bern, Bern, Switzerland; 2 Faculty of Medicine, Masaryk University, Brno, Czech Republic; 3 Institute of Radiology, Cantonal Hospital Münsterlingen, Münsterlingen, Switzerland; 4 Department of Otorhinolaryngology, Head and Neck Surgery, Inselspital, Bern University Hospital and University of Bern, Bern, Switzerland; 5 Department of Neurology, Inselspital, Bern University Hospital, University of Bern, Bern, Switzerland; 6 Department of Neurology, Medical School, University of Cyprus, Nicosia, Cyprus; 7 Department of Emergency Medicine, Inselspital, Bern University Hospital and University of Bern, Bern, Switzerland

**Keywords:** Styloid process, Internal carotid artery, Dissection, Stroke

## Abstract

**Purpose:**

Internal carotid artery dissection (ICA-D) frequently leads to ischemic stroke in individuals under 50 years. There is mounting evidence on the role of the styloid process (SP) in ICA-D, particularly SP length and the SP–ICA distance. Despite having clear guidelines on the treatment of SP-related Eagle syndrome and ICA-D, the concept of SP-related ICA-D is relatively new and no therapeutic guidelines exist.

**Methods:**

A narrative literature search was performed to identify all articles pertaining to the diagnosis and treatment of ICA-D linked to an ipsilateral elongated SP or short SP-ICA distance. The treatments were evaluated in terms of symptom recurrence after the treatment. As illustrative examples of clinical management, we present an in-house case series of patients with suspected ICA-D related to SP.

**Results:**

Treatment efficacy was assessed, with an in-house case series provided. Seventy-five reports and case studies involving 84 patients were analyzed. Conservative treatments were common (52%) but had a high symptom recurrence rate (33%). It is noteworthy that no patients treated initially with styloidectomy exhibited symptom recurrence.

**Conclusion:**

In case of a correctly diagnosed SP-related ICA-D, a styloidectomy may offer a curative option, but more research is needed for clear indications and standardized guidelines to prevent recurrent symptoms or strokes.

**Supplementary Information:**

The online version contains supplementary material available at 10.1007/s00234-025-03616-y.

## Introduction

**Fig. 1:**
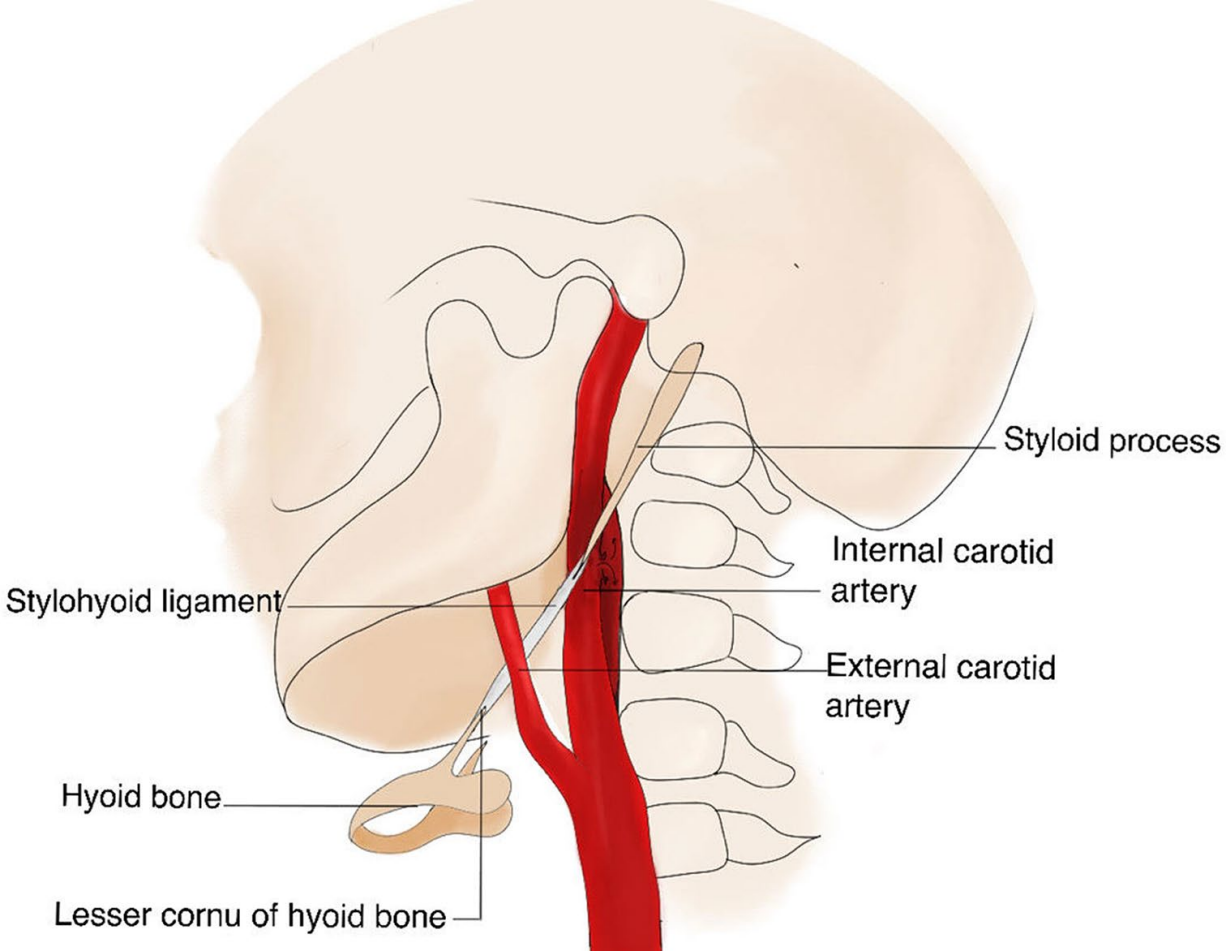
Illustration of the anatomical relations of the elongated SP causing damage to the wall of the cervical ICA. SP– styloid process, ICA– internal carotid artery

Internal carotid artery dissection (ICA-D) is a common cause of ischemic stroke, especially in patients under the age of 50 years. The majority of these patients present with of spontaneous dissections [1]. An elongated styloid process (SP), defined as an SP exceeding 30 mm in length, is present in about 30% of the population and is known to have many anatomical variations [2, 3]. The anatomical relationship of an elongated SP with damage to the wall of the cervical ICA is illustrated in Fig. 1. Since the first case report was published 1999, presenting a patient with ICA-D due to an elongated SP [4], mounting evidence on this condition has been published, not only in the form of case reports, but also case-cohort studies and a meta-analysis [5–9]. However, to the best of our knowledge, the literature available so far does not cover the available sources of evidence and does not focus on the optimal therapeutic approach [10–12].

## Methods

The aim of this study was to provide an up-to-date narrative review of the available literature on ICA-D linked to an elongated SP or short SP-ICA distance, paying particular attention to the options available for treatment of patients with ICA-D. We provide a series of cases from our own institution as illustrative examples of different treatment approaches. By establishing further guidelines emphasizing the role of prophylactic elective styloidectomy in patients with ICA-D related to SP, the rate of recurrent symptoms or ischemic events could be minimized, especially in younger people.

## Case series

### Case 1

#### Clinical history

**Fig. 2:**
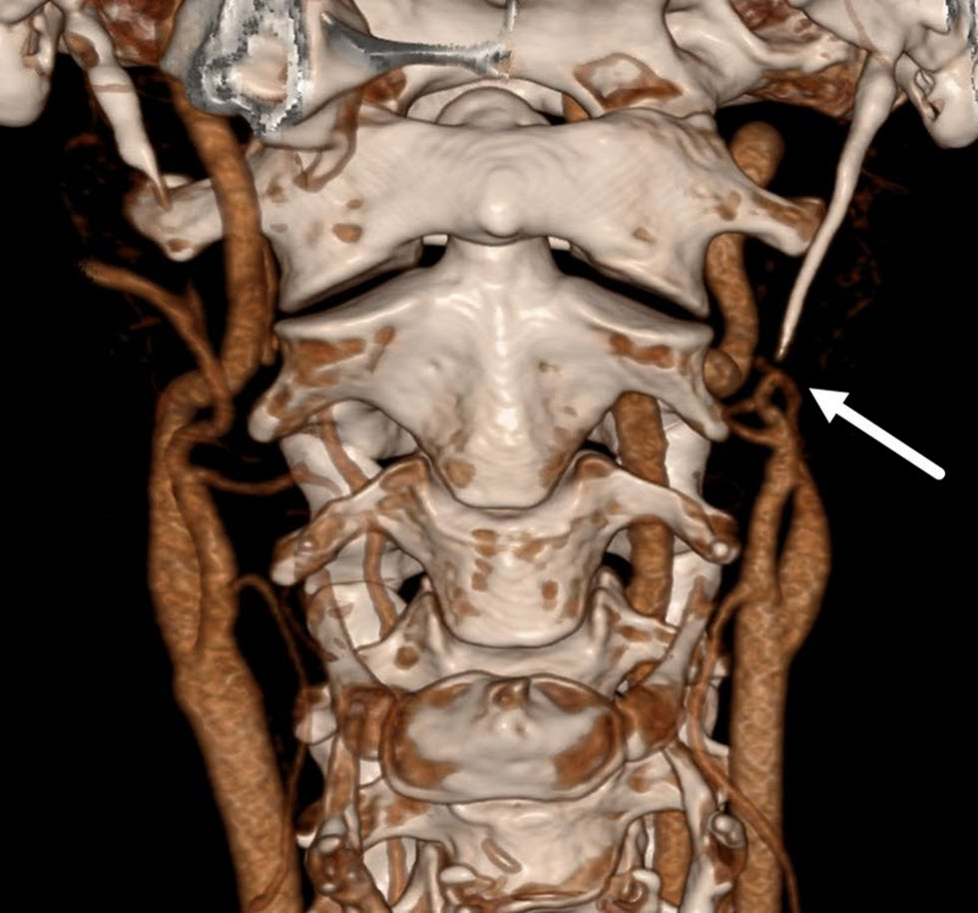
3D reconstruction of the CT-angiography shows a complete occlusion of the left ICA starting from the point of contact with the tip of the SP (white arrow). CT– computed tomography, ICA– internal carotid artery, SP– styloid process

A 50-year-old female patient with no significant medical history presented at our emergency department (ED) complaining of progressive headaches over the last couple of days, and most recently, also in the mandibular region, after cleaning the swimming pool. An initial neurologic exam demonstrated left-sided Horner’s syndrome. An immediately-initiated MRI scan showed a long-segment dissection of the cervical and petrous segment of the left ICA. A subsequent CTA confirmed the ICA-D with collapse of the true lumen in the petrous segment (Fig. 2). The CTA also revealed an elongated SP on the ipsilateral side of the ICA-D (left: 34 mm, right: 22 mm). The patient was treated with aspirin and discharged after 3 days due to complete symptom remission. Two weeks after the initial symptoms, the patient presented again at our ED with recurrence of left-sided Horner’s syndrome. Another MRI was performed immediately. This showed a progressive collapse of the entire cervical and petrous ICA lumen on the left side. After an interdisciplinary case discussion, elective surgical removal of the left SP via a transcervical approach was performed. At the follow-up visit at our hospital after the styloidectomy, the patient remained symptom-free.

#### Surgical procedure

**Fig. 3:**
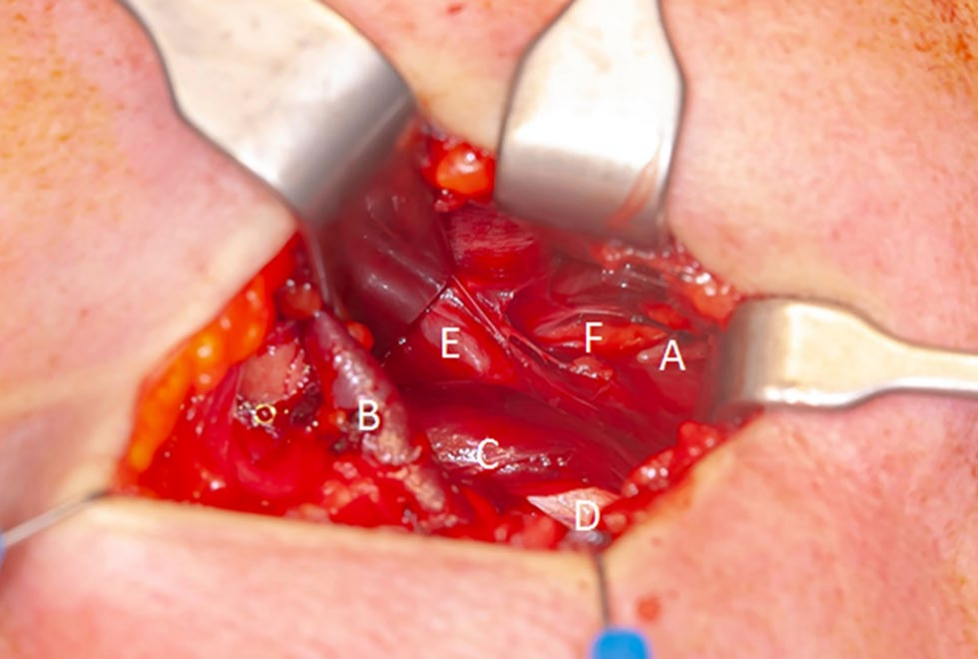
Intraoperative image during prophylactic styloidectomy. A– tip of the styloid process, B - Facial vein, C - Stylohyoid muscle, D - Tendon of the digastric muscle, E - Facial artery, F - Thrombosed ICA. ICA– internal carotid artery

Under general anesthesia, a transverse incision of approximately 3 cm was made below the lower border of the angle of the mandibula. A blunt dissection and exposure of the posterior belly of the digastric muscle and the mylohyoid muscle was performed. The left SP was identified just lateral to the ICA, which was pulseless due to due to thrombosis from the dissection (shown in Fig. 3). The SP was freed and shortened by 1.7 cm with a diamond drill.

### Case 2

A 40-year-old female patient with a medical history of episodic migraine presented at our ED with acute onset of a right-sided headache and pulsatile tinnitus in the right ear. The patient reported no recent trauma or intense physical activity involving neck movements. No neurological deficits were observed. Based on the symptoms, the neurologists suspected an ICA-D and a confirmatory MRI of the head and neck was performed on the same day. This MRI showed a long-segment dissection on the cervical segment of the right ICA with an adjacent pseudoaneurysm. On a native CT scan 9 years before the event, which had been performed for chronic sinusitis, an elongated SP was identified on the right side (right: 40 mm; left: 24 mm). The SP tip was located directly adjacent to the location of the ICA-D. The patient was started on long-term aspirin therapy. After the diagnosis, the patients declined surgical or endovascular interventions. At the 1-year follow-up, there was a partial regression of the initial symptoms with residual headaches. In follow-up MRI the dissection of the cervical ICA was unchanged, showing the pseudoaneurysm but no vessel-wall hematoma or significant stenosis of the ICA. No ischemic events or other complications were observed.

### Case 3

**Fig. 4:**
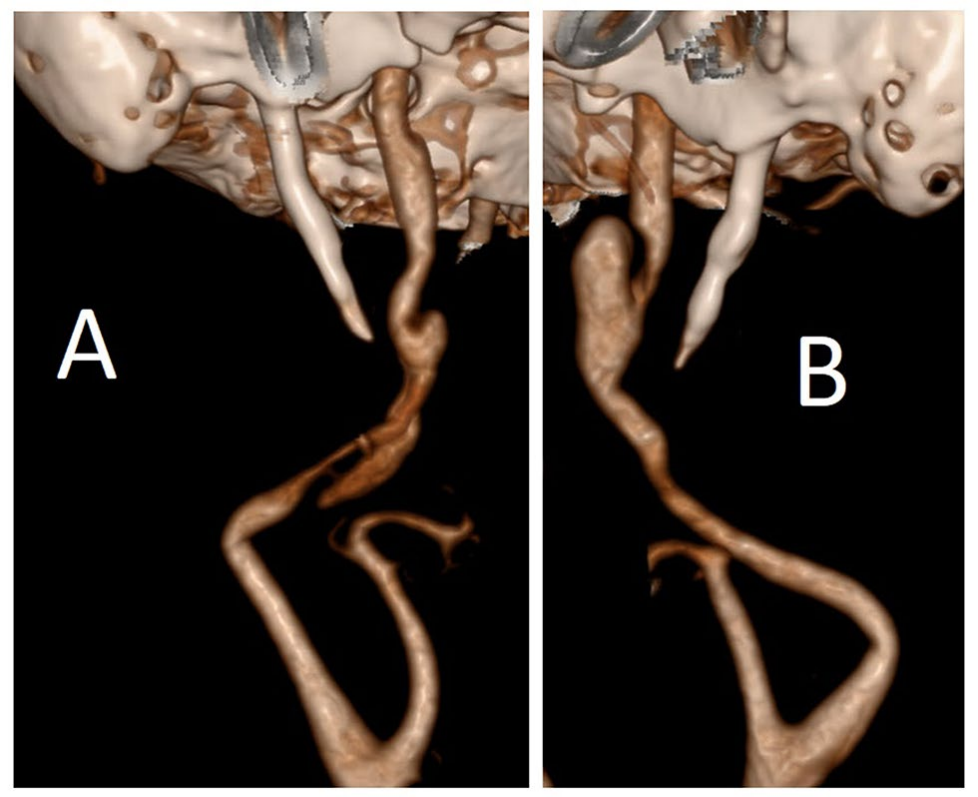
3D reconstruction of the CT-angiography demonstrating the ICA-D of the right (**A**) and left (**B**) side with short SP-ICA distance. CT– computed tomography, ICA-D– internal carotid artery dissection, ICA– internal carotid artery, SP– styloid process

A 70-year-old female patient complaining of paresthesia and weakness on the left side of her body presented to the ED of an external hospital. No relevant medical history was reported. The patient reported no previous interventions in the neck region, traumas or intense sporting activities. An initial CTA was performed, since no MRI facilities were available at the external center. The CTA showed bilateral ICA-D in the cervical segment, alongside a bilaterally elongated SP (right: 39 mm, left: 32 mm), see Fig. 4. Because the initial finding did not provide a clear explanation for the patient’s symptoms, the patient was referred to our institute, where an MRI exam was performed, demonstrating a small ischemic lesion in the left paramedian pons region, not related to the ICA-Ds. Aspirin was administered as part of stroke therapy. No further therapeutic steps were taken since the ICA-Ds did not cause a high-grade stenosis or a potentially serious perfusion deficit in the brain. The patient was discharged home without any neurological deficits or complaints. In the follow-up examination 3 months after the event, the patient remained asymptomatic.

## Overview of patient demographics from the search of case reports and series

**Fig. 5:**
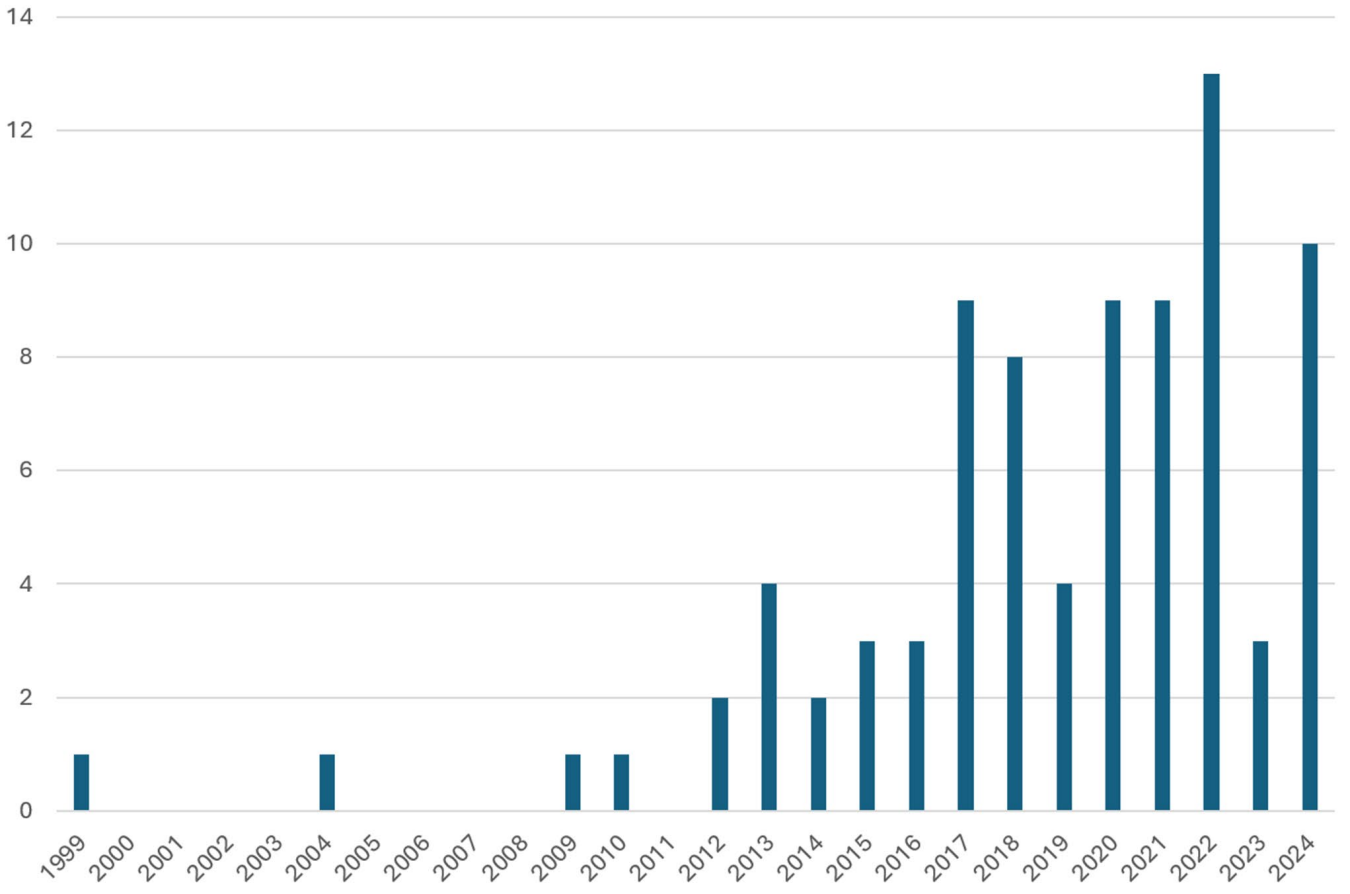
Graphical representation of the number of cases published per year on the topic of ICA-D due to SP until 2024 (our own case series is included in the year 2024). ICA-D– internal carotid artery dissection, SP– styloid process

After excluding ineligible studies, our literature search in the PubMed and SCOPUS databases identified a total of 75 reported studies. A total of 84 patients were included together with our own 3-patient case series (Supplementary Table 1) Fig. 5.

According to our literature review, ICA-D seems to affect mainly middle-aged patients (median age: 47) and predominates in men (77%). No causative events directly preceding the ICA-D were reported in the majority of the patients (81%). However, a substantial number of patients (14%) reported abrupt and/or repetitive neck movements or manipulation of the neck [13–23], prompting us to put more focus on such events during history taking. Four patients (5%) have had a direct trauma preceding the hospital admission directly responsible for the ICA-D [24–26]. In one patient, ICA-Ds was associated with a surgical intervention [27]. The demographics of all patients included in our analysis of the case reports and series, together with those of our 3 patients, are summarized in Table 1.

**Table 1:** Demographics of patients included in the identified case reports and series including our 3 patients

	Overall	Missing
**N**	87	
**Age (median [IQR])**	47 [41–55]	3 (3.4)
**Male/female**,** n (%)**	67/20 (77/23)	0 (0)
**Trauma/activity/surgery directly preceding the ICA-D**,** n (%)**		0 (0)
No	70 (80.5)	
Trauma	4 (4.6)	
Activity	12 (13.8)	
Iatrogenic	1 (1.1)	
**Side of the dissection**,** n (%)**		0 (0)
Right	32 (36.8)	
Left	35 (40.2)	
Bilateral	20 (23.0)	
**SP length on the right side**,** mm (median [IQR])**	39 [32–47]	38 (43.7)
**SP length on the left side**,** mm (median [IQR])**	39 [32–49]	41 (47.1)
**SP length on the affected side**,** mm (median [IQR])**	37 [32–49]	29 (33.3)
**Symptomatic on admission**,** n (%)**		1 (1.1)
No	3 (3.4)	
Yes	83 (95.4)	
**Neurological deficits at admission**,** n (%)**		1 (1.1)
No	11 (12.6)	
Yes	75 (86.2)	

N– number, IQR– interquartile range; SP– styloid process

The SP on the affected side was elongated in all the cases presented, except for 2 patients where the SP length was 29 mm. This is in agreement with the findings of a later meta-analysis of the published case-control studies, which reported a significant relationship between the SP length and the rate of ICA-Ds [28]. However, the number of missing data on SP length was very high (33%) and more detailed reports would be needed to confirm these findings. Other potential confounders (e.g., SP-angulation, SP–ICA distance, SP-type, etc.) were seldom reported. However, studies published since 2025 have been more consistent in reporting the SP-ICA distance [29–31]. Although most patients (86%) showed symptoms and neurological deficits on admission, in 3% of patients the ICA-D related to SP was detected incidentally with chronic changes to the ICA-wall and related development of pseudoaneurysms [10, 32]. This leads us to consider the SP not only as a cause of acute ICA-D, but also in terms of the chronic changes it may cause in its immediate surroundings. In 3 cases, contact with the SP tip caused a fracture of a previously implanted stent [21, 33, 34].

### Diagnosis

After clinical examination, which mostly identifies neurological deficits (most commonly: hemiparesis, Horner’s syndrome, aphasia, etc.), the imaging modality of choice is a CTA. This is because a CTA can not only demonstrate ICA stenosis but also allows an assessment of the anatomy of the SP (length, angulation or type according to Langlais [35]) and its relationship with the ICA (SP–ICA distance, SP tip–ICA distance). An MRI exam may be helpful in the visualization of the arterial wall and any intramural hematoma [36].

Currently, there are no internationally standardized guidelines or criteria for establishing the diagnosis of an ICA-D due to SP and the pathology is still not widely recognized as a causative factor of ICA-D [36, 37]. Therefore, the diagnosis is often left to the discretion of the individual radiologist. The most commonly reported characteristic of the SP in the case reports was its length. In 2023, a meta-analysis from England analyzed the SP length of 185 patients with ICA-D and 278 controls. After sensitivity analysis, it was confirmed that ICA-D is significantly associated with SP mean length and an SP length > 30 mm [28]. However, most of the studies analyzed had a small sample size, the methodology varied, and many did not distinguish between spontaneous and traumatic ICA-D. Other factors (e.g. SP–ICA distance) need to be reported routinely to better understand the pathological mechanism leading to the dissection.

### Treatment

There being no standardized guidelines for ICA-D due to elongated SP, the treatment decision was left to the respective clinician, resulting in differing initial treatment strategies. All treatment characteristics are summarized in Table 2.

**Table 2:** Treatment strategy in the 80 patients from the case reports and case series together with our 3 patients

	Overall	Missing
**N**	87	
**Initial treatment**,** n (%)**		4 (4.6)
None	4 (4.6)	
Antiplatelets	21 (24.1)	
Anticoagulants	17 (19.5)	
Carotid artery stenting	6 (6.9)	
Styloidectomy	6 (6.9)	
Antiplatelets + anticoagulants	7 (8.0)	
Antiplatelets + carotid artery stenting	10 (11.5)	
Antiplatelets + anticoagulants + carotid artery stenting	5 (5.7)	
Styloidectomy + anticoagulants	5 (5.7)	
Styloidectomy + antiplatelets	1 (1.1)	
Carotid artery stenting + anticoagulants	1 (1.1)	
**Symptom recurrence**,** n (%)**		13 (14.9)
No	53 (60.9)	
Yes	21 (24.1)	
**Additional treatment**,** n (%)**		6 (6.9)
None	47 (54.0)	
Carotid artery stenting	9 (10.3)	
Styloidectomy	18 (20.7)	
Styloidectomy + Carotid artery stenting	4 (4.6)	
Antiplatelets	2 (2.3)	
Antiplatelets + Embolisation	1 (1.1)	
**Symptom recurrence after additional treatment**,** n (%)**		13 (14.9)
No	25 (28.7)	
Yes	1 (1.1)	
Not applicable	48 (55.2)	

N - number

#### Conservative therapy

Most ICA-Ds heal spontaneously. To prevent complications in the form of thromboembolic events, antiplatelet or anticoagulation therapy is usually initiated [36, 37]. In most of the cases presented, non-invasive or conservative treatment, usually consisting of antiplatelet (24%) [16, 18, 34, 38–51] or anticoagulant (20%) [4, 14, 19, 52–61] agents separately or combined (8%) [21, 25, 61–65], was the first-line option. Out of all 20 patients with symptom recurrence (complete or partial) after initial treatment (23%), 71% had been treated conservatively. This prompts us to consider and discuss other treatment alternatives (Supplementary Table 2).

#### Carotid artery stenting

Twenty-two patients (25%) were initially treated with carotid artery stenting, alone or combined with antiplatelets or anticoagulants [10–13, 15, 17, 24, 27, 29–31, 34, 66–73]. Five of these patients (24%) experienced symptom recurrence [13, 29, 34, 71, 73]. Nowadays, endovascular treatment in ICA-D patients is less often indicated because of the low rate of recurrence, the risk of peri- and postprocedural thromboembolic events, which could potentially be reduced by the use of double-layer stents [36, 74], and the risk of acute in-stent thrombosis [68]. Two cases have been reported where the continuous compression by the SP caused a stent fracture [21, 33].

In the context of prolonged contact of the ICA with the SP, the stent may serve as a protective shield that prevents further arterial stenosis. However, regardless of stent design, the arterial wall may still be irritated by the continuous contact with the SP, depending on its characteristics.

#### Surgical treatment

Styloidectomy, alone or combined with antiplatelets or anticoagulants, was performed as part of the initial treatment in 12 patients (14%) [10, 20, 22, 32, 61, 75–79] and it was performed as part of the additional treatment in 69% of patients with symptom recurrence. The fact that no patients treated with styloidectomy suffered symptom recurrence after initial treatment raises the question of the value of such a preventive operation. Furthermore, only one patient showed symptom recurrence in the form of headaches after additional treatment with styloidectomy [69]. So far, the number of patients with ICA-D due to SP treated with styloidectomy has not been sufficient to prove its efficacy. Larger randomized controlled trials are needed.

Styloidectomy was first introduced by Watt Weems Eagle in the 1940s [80]. The standard approaches used today are either intraoral or transcervical. The intraoral approach is reported to be quicker and simpler but is now used less frequently due to poorer visualization of the surgical field and an association with more complications, such as bleeding and retropharyngeal infection [19, 44, 54, 63, 71, 81–83]. Some studies have employed successfully modified techniques to reduce these risks, showing good results in small groups of patients [84]. Endoscopic or da Vinci robotic surgery has also been explored but has not yet been widely adopted [85–88].

The more commonly preferred transcervical approach, which we also chose, provides a better view and access to the SP and surrounding areas, making it safer for delicate work around the ICA, especially in a dissection [89, 90]. This approach has proven to be safe and effective, with few complications reported even in larger groups of patients. In a larger case study of 61 patients, only 2 had temporary marginal mandibular nerve weakness, with no other reported complications, supporting the safety and efficacy of this technique [91].

Styloidectomy remains a critical intervention for patients with recurrent ICA-D related to the styloid process. Patients with an elongated SP are prone to have a shorter SP-ICA distance [92]. Evidence indicates that patients with significant anatomical conflict between the SP and the ICA, as demonstrated by imaging studies, benefit most from this procedure [93, 94]. This is particularly relevant in cases where conservative and endovascular treatments fail to prevent symptom recurrence, as suggested by the low recurrence rates observed after styloidectomy. Ultimately, it remains the primary treatment option to resolve the mechanical conflict between the ICA and the elongated SP. The available evidence strongly supports favoring styloidectomy in cases of neurological deterioration despite adequate conservative and endovascular treatment, as demonstrated in one of our cases. Another recent study suggests basing the criterion for styloidectomy on the severity of the clinical case [95]. Additionally, styloidectomy is recommended during a stable, chronic stage of the disease or in cases with bilateral elongated SP and unilateral ICA-D to prevent recurrence and should be discussed with the patient in the clinical setting [13, 29, 60, 61, 67].

## Study limitations

This study has some limitations. Despite using a relatively broad approach to the identification of eligible publications, it is possible that some reports that did not meet the selection criteria but included relevant information were inadvertently excluded. Moreover, the risk of bias was not assessed because we deemed it necessary to include all available information in our review. The heterogeneity of the reports included and their omission of some important data (e.g., SP length on the affected side and symptom recurrence) led to difficulty in analyzing the results. While we acknowledge the sample size may appear small, we consider it a particular strength of this review that such a number of cases could be gathered, given the rarity of the disease. This provides a unique and valuable opportunity to analyze a relatively large cohort in this specific context. Future reports should systematically include all relevant patient data (e.g. SP length, SP–ICA distance, symptom recurrence, comorbidities and other risk factors, treatment strategy, etc.).

## Conclusion

Despite mounting evidence supporting the association of ICA-D with SP elongation, the correct and early diagnosis by neuroradiologists is key to establish optimal further clinical management and treatment of patients with SP elongation. However, its causal role and the best therapeutic strategies have yet to be conclusively established. Large-scale case–control studies are warranted to define the precise relationship between SP variants and ICA-D, and their impact on the incidence of ICA-Ds. Such endeavors would not only enhance physicians’ awareness of SP-induced ICA-D but also offer potential for styloidectomy as an intervention in both healthy individuals and those with ICA-D, thereby mitigating the occurrence and prevalence of ICA-Ds and associated ischemic events.

## Electronic supplementary material

Below is the link to the electronic supplementary material.

Supplementary Material 1

Supplementary Material 2

## Data Availability

Data are available upon reasonable request.

## Abbreviations

ED: emergency department
ICA-D: internal carotid artery dissection
SP: styloid process
ST: Styloidectomy
