# Supplementary material for: Styloid process-related internal carotid artery dissection: extensive literature review of diagnosis, treatment and outcomes (exemplified with a single-center case series)
Source: Neuroradiology. 2025 Apr 26;67(6):1355–64. doi: 10.1007/s00234-025-03616-y (PMC12357798; doi:10.1007/s00234-025-03616-y)
Supplement: Supplementary file 1 — Supplementary Material 1 [file 234_2025_3616_MOESM1_ESM.docx]

**Supplementary materials**

*Styloid Process-Related Internal Carotid Artery Dissection:*

*Extensive literature review of diagnosis, treatment and outcomes (exemplified with a single-center case series)*

**Supplementary Table 1 - Reported cases of ICA-D related to SP included in our review**

| **Author** | **Year** | **Age** | **Sex** | **Previous trauma/ activity/surgery** | **Symptoms on admission** |
| --- | --- | --- | --- | --- | --- |
| Zuber et al. | 1999 | 43 | M | None reported | Ocular TIA, aphasia |
| Soo et al. | 2004 | 41 | F | None reported | Ocular TIA |
| Faivre et al. | 2009 | 60 | M | None reported | Hemiparesis, Horner's syndrome, hemianopsia |
| Cano et al. | 2010 | 45 | F | None reported | Neck pain, hemiparesis, dysarthria |
| Ohara et al. | 2012 | 43 | M | None reported | Neck pain, dysarthria |
| Todo et al. | 2012 | 57 | M | None reported | Transient aphasia, neck pain |
| Sveinsson et al. | 2013 | 38 | M | None reported | Hemiparesis, dysarthria |
| Sveinsson et al. | 2013 | 41 | F | Activity | Headache |
| Yamamoto et al. | 2013 | 51 | M | None reported | Right orbital pain, Horner's syndrome |
| Vodopivec et al. | 2013 | 52 | M | None reported | Headache, diplopia |
| Naito et al. | 2014 | 55 | M | None reported | Dizziness, visual loss |
| Razak et al. | 2014 | 41 | M | Activity | Left hemiparesis, left visual field deficit |
| Ogura et al. | 2015 | 55 | M | None reported | Left sided amaurosis fugax |
| Ogura et al. | 2015 | 55 | M | None reported | Left sided hemiplegia |
| Ogura et al. | 2015 | 80 | M | None reported | Odynophagia, positional vertigo |
| Dewan et al. | 2016 | 49 | M | None reported | Right facial weekness, partial aphasia |
| Hooker et al. | 2016 | 64 | M | None reported | None |
| Miyata et al. | 2016 | 41 | M | None reported | Left hemidysesthesia |
| Mann et al. | 2017 | 38 | M | Trauma | Dysarthria, neck pain |
| Mann et al. | 2017 | 39 | F | Trauma | Left hemiparesis |
| Jo et al. | 2017 | 38 | F | None reported | Headache, left perioral area numbness |
| Hebant et al. | 2017 | 57 | M | None reported | Aphasia, facial palsy |
| Amorim et al. | 2017 | 68 | M | None reported | Aphasia, facial palsy, headache |
| Subedi et al. | 2017 | 47 | F | None reported | Aphasia, left arm weakness |
| Smoot et al. | 2017 | 60 | M | None reported | Headache, left arm and leg paresthesia |
| Langlet et al. | 2017 | 36 | F | None reported | Right neck mass |
| Aldakkan et al. | 2017 | 85 | M | None reported | Aphasia, right hemiparesis, right facial droop |
| Aydin et al. | 2018 | 57 | F | None reported | Dizziness, sightedness |
| Jelodar et al. | 2018 | 40 | M | None reported | Right hemiparesis and dysphasia |
| Higashi et al. | 2018 | 45 | F | None reported | Pharyngalgia, neck pain |
| Shimozato et al. | 2018 | 48 | M | Activity | Incomplete left hemiplegia |
| Shimozato et al. | 2018 | 45 | M | None reported | Aphasia, paralysis right limb |
| Takino et al. | 2018 | 46 | M | None reported | Aphasia, left visual disturbance |
| Sullivan et al. | 2018 | 62 | M | None reported | Right -sided weakness and behavioral changes |
| Zammit et al. | 2018 | 45 | M | None reported | Right Horner's syndrome, headache |
| Shindo et al. | 2019 | 49 | M | None reported | Left hemiplegia, dysarthria |
| Yano et al. | 2019 | 58 | M | None reported | Right paresthesia, aphasia |
| Quershi et al. | 2019 | 42 | F | Activity | Right hemiparesis, facial weakness, aphasia |
| Torikoshi et al. | 2019 | 46 | M | None reported | Left hemiplegia, reduced consciousness, vomiting |
| Baldino el al. | 2020 | 41 | F | None reported | None |
| Baldino el al. | 2020 | 48 | M | None reported | Right transient hemiparesis |
| Baldino el al. | 2020 | 45 | M | None reported | Left amaurosis fugax |
| Baldino el al. | 2020 | 49 | M | None reported | Aphasia |
| Baldino el al. | 2020 | 56 | M | None reported | Transient loss of consciousness |
| Horio et al. | 2020 | 46 | F | None reported | Right hemiparesis and aphasia |
| Ikenouchi et al. | 2020 | 30 | M | None reported | Mild sensory disturbance in left limbs |
| Kesav et al. | 2020 | 49 | M | None reported | Aphasia and right hemiparesis |
| Bassart et al. | 2020 | 53 | F | None reported | Left hemiplegia, tinnitus |
| Tanti et al. | 2021 | Young | M | Activity | Headache, left- sided facial weakness, visual extinction |
| AlAbdulwahed et al. | 2021 | 35 | M | None reported | Left arm weakness, facial droop |
| Yokoya et al. | 2021 | 59 | M | None reported | Amaurosis fugax left |
| Xhaxho et al. | 2021 | 64 | M | None reported | Loss of conciousness, headache, right amaurosis |
| Duarte-Celada et al. | 2021 | 43 | F | None reported | Headache, pharyngalgia, left-sided Horner's syndrome |
| Mattioli et al. | 2021 | 56 | M | None reported | Loss of consciousness, confusion, dysarthria |
| Baez-Martinez et al. | 2021 | 36 | M | Activity | Headache, left-sided Horner's syndrome |
| Abe et al. | 2021 | 51 | M | None reported | Motor weakness, numbness in right arm |
| Ramadan | 2021 | 47 | M | None reported | Left extremity weakness, left sensory loss, dysarthria |
| Hayashi et al. | 2022 | 69 | M | None reported | Right hemiplegia, aphasia |
| Selvadurai et al. | 2022 | Teen | M | Activity | Left hemiparesis, dysarthria and headache |
| Shi et al. | 2022 | 30 | M | None reported | Left sensorimotor deficit |
| Lakner et al. | 2022 | 38 | M | None reported | Right hemiparesis, aphasia |
| Sasaki et al. | 2022 | 37 | M | None reported | Right amaurosis fugax |
| Okada et al. | 2022 | 72 | M | None reported | Dysarthria and consciousness disorder |
| Suzuki et al. | 2022 | 69 | M | Iatrogenic | Right hemiparesis and dysarthria |
| Rocco et al. | 2022 | 21 | M | None reported | None |
| Gomez et al. | 2022 | 48 | M | None reported | Right-sided Horner's syndrome |
| Diaz Cruz et al. | 2022 | 37 | M | Activity | Headache, ptosis |
| Matsukawa et al. | 2022 | 38 | M | None reported | Aphasia, right hemiplegia |
| Pfaff et al. | 2022 | 40s | M | Activity | Ptosis, left eye visual disturbance |
| Pagano et al. | 2022 | 48 | 0 | None reported | Massive enoral bleeing, desorientation |
| Berrada et al. | 2023 | 40 | M | None reported | Facial paralysis, left sensorimotor deficit |
| Putek et al. | 2023 | 36 | F | Trauma | Left body weakness, facial palsy, dysarthria |
| Sarwar et al. | 2023 | 57 | M | None reported | Dysarthria, left-sided body weakness |
| Zheng et al. | 2024 | 63 | M | Trauma | Dysphagia |
| Obara et al. | 2024 | 47 | F | None reported | Headache |
| Luo et al. | 2024 | 50 | M | None reported | Dizziness, left limb fatigue |
| Sayed et al. | 2024 | 22 | M | Activity | Torticollis, epistaxis |
| Andereggen et al. | 2024 | 37 | F | None reported | Headache, neck pain, Horner's syndrome |
| Safan et al. | 2024 | 44 | M | None reported | Headache, left hemiparesis, dysarthria |
| Bargiel et al. | 2024 | 43 | M | Activity | Dysarthria, right arm weakness |
| Present case | 2024 | 40 | F | None reported | Flow-noice, right-sided headache |
| Present case | 2024 | 50 | F | None reported | Headache and left-sided Horner's syndrome |
| Present case | 2024 | 70 | F | None reported | Retroauricular pain, headache |
| Vigilante et al. | 2025 | 48 | M | None reported | Aphasia and arm weakness |
| Osuki et al. | 2025 | 43 | M | None reported | Left hemiparesis, left hemispatial neglect and dysarthria |
| Vos et al. | 2025 | 44 | M | Activity | Left arm paresis, head and neck pain, left facial nerve paralysis |
| Hopf-Jensen et al. | 2025 | 53 | M | None reported | Aphasia and right-sided hemiplegia |

ICA-D – internal carotid artery dissection, SP – styloid process

**Supplementary Table 2 – Patient baseline data and initial therapy stratified by symptom recurrence after initial therapy**

|  | **Overall** | **No** | **Yes** | **p-value** | **Missing** |
| --- | --- | --- | --- | --- | --- |
| **N** | 87 | 53 | 21 |  |  |
| **Age (median [IQR])** | 46 [41 - 55] | 47 [41 - 55] | 48 [41 - 56] | 0.995 | 3 (3.4) |
| **Male/female, n (%)** | 67/20 (77.0/23.0) | 38/15 (71.7/28.3) | 17/4 (81.0/19.0) | 0.599 | 0 (0.0) |
| **Trauma/activity/surgery directly preceding the ICA-D, n (%)** |  |  |  | 0.788 | 0 (0.0) |
| No | 70 (80.5) | 41 (77.4) | 18 (85.7) |  |  |
| Trauma | 4 (4.6) | 2 (3.8) | 1 (4.8) |  |  |
| Activity | 12 (13.8) | 9 (17.0) | 2 (9.5) |  |  |
| Iatrogenic | 1 (1.1) | 1 (1.9) | 0 (0.0) |  |  |
| **SP length on the affected side, mm (median [IQR])** | 37 [32 - 49] | 36 [32 - 50] | 42 [36 - 49] | 0.399 | 28 (33.3) |
| **Initial treatment, n (%)** |  |  |  | NS | 4 (4.6) |
| None | 4 (4.6) | 1 (1.9) | 1 (4.8) |  |  |
| Antiplatelets | 21 (24.1) | 11 (20.8) | 8 (38.1) |  |  |
| Anticoagulants | 17 (19.5) | 12 (22.6) | 4 (19.0) |  |  |
| Carotid artery stenting | 6 (6.9) | 4 (7.5) | 2 (9.5) |  |  |
| Styloidectomy | 6 (6.9) | 5 (9.4) | 0 (0.0) |  |  |
| Antiplatelets + anticoagulants | 7 (8.0) | 3 (5.7) | 3 (14.3) |  |  |
| Antiplatelets + carotid artery stenting | 10 (11.5) | 6 (11.3) | 3 (14.3) |  |  |
| Antiplatelets + anticoagulants + carotid artery stenting | 5 (5.7) | 4 (7.5) | 0 (0.0) |  |  |
| Styloidectomy + anticoagulants | 5 (5.7) | 5 (9.4) | 0 (0.0) |  |  |
| Styloidectomy + antiplatelets | 1 (1.1) | 1 (1.9) | 0 (0.0) |  |  |
| Carotid artery stenting + anticoagulants | 1 (1.1) | 1 (1.9) | 0 (0.0) |  |  |

N – number, NS – not significant, IQR – interquartile ratio, n – number, SP – styloid process, APT – antiplatelets, ACG – anticoagulants, CAS – carotid artery stenting,

**Supplementary Table 3 – Patient baseline data and additional therapy stratified by symptom recurrence after additional therapy**

|  | **Overall** | **No** | **Yes** | **Not applicable** | **Missing** |
| --- | --- | --- | --- | --- | --- |
| **N** | 87 | 25 | 1 | 48 |  |
| **Age (median [IQR])** | 47 [41 - 55] | 48 [42 - 55] | 43 [43 - 43] | NA | 3 (3.4) |
| **Male/female, n (%)** | 67/20 (77.0/23.0) | 21/4 (84.0/16.0) | 0/1 (0.0/100.0) | NA | 0 (0.0) |
| **Trauma/activity/surgery directly preceding the ICA-D, n (%)** |  |  |  | NA | 0 (0.0) |
| No | 70 (80.5) | 22 (88.0) | 1 (100.0) |  |  |
| Trauma | 4 (4.6) | 1 (4.0) | 0 (0.0) |  |  |
| Activity | 12 (13.8) | 2 (8.0) | 0 (0.0) |  |  |
| Iatrogenic | 1 (1.1) | 0 (0.0) | 0 (0.0) |  |  |
| **SP length on the affected side, mm (median [IQR])** | 37 [32 - 49] | 35 [32 - 51] | NA | NA | 29 (33.3) |
| **Additional treatment, n (%)** |  |  |  | NA | 6 (6.9) |
| None | 47 (54.0) | 0 (0.0) | 0 (0.0) |  |  |
| Carotid artery stenting | 9 (10.3) | 7 (28.0) | 0 (0.0) |  |  |
| Styloidectomy | 18 (20.7) | 13 (52.0) | 1 (100.0) |  |  |
| Styloidectomy + carotid artery stenting | 4 (4.6) | 4 (16.0) | 0 (0.0) |  |  |
| Antiplatelets + embolisation | 2 (2.3) | 0 (0.0) | 0 (0.0) |  |  |
| Antiplatelets | 1 (1.1) | 1 (4.0) | 0 (0.0) |  |  |

N – number, NA – not applicable, SP – styloid process, IQR – interquartile ratio
